# The cochlear ear horn: geometric origin of tonotopic variations in auditory signal processing

**DOI:** 10.1038/s41598-020-77042-w

**Published:** 2020-11-25

**Authors:** Alessandro Altoè, Christopher A. Shera

**Affiliations:** 1 Caruso Department of Otolaryngology, University of Southern California, Los Angeles, CA USA; 2 Department of Physics and Astronomy, University of Southern California, Los Angeles, CA USA

**Keywords:** Biological physics, Sensory processing

## Abstract

While separating sounds into frequency components and subsequently converting them into patterns of neural firing, the mammalian cochlea processes signal components in ways that depend strongly on frequency. Indeed, both the temporal structure of the response to transient stimuli and the sharpness of frequency tuning differ dramatically between the apical and basal (i.e., the low- and high-frequency) regions of the cochlea. Although the mechanisms that give rise to these pronounced differences remain incompletely understood, they are generally attributed to tonotopic variations in the constituent hair cells or cytoarchitecture of the organ of Corti. As counterpoint to this view, we present a general acoustic treatment of the horn-like geometry of the cochlea, accompanied by a simple 3-D model to elucidate the theoretical predictions. We show that the main apical/basal functional differences can be accounted for by the known spatial gradients of cochlear dimensions, without the need to invoke mechanical specializations of the sensory tissue. Furthermore, our analysis demonstrates that through its functional resemblance to an ear horn (aka ear trumpet), the geometry of the cochlear duct manifests tapering symmetry, a felicitous design principle that may have evolved not only to aid the analysis of natural sounds but to enhance the sensitivity of hearing.

## Introduction

To facilitate the detection and analysis of sound, the mammalian cochlea acts as an acoustic prism, mapping sound frequency onto position and thereby onto different populations of sensory cells. Mechanical and neural responses reveal that the characteristics of the inner ear’s nonlinear signal processing vary systematically with tonotopic location along the cochlear spiral. Indeed, differences between the apical and basal halves of the cochlea—where responses are tuned to low and high frequencies, respectively—are striking. Whereas frequency responses are sharply tuned and nearly scaling symmetric in the base, they become substantially broader and more complex in the apex^1–3^. Temporal features of the response to transient stimuli, such as acoustic clicks, also differ qualitatively. In the base, the instantaneous frequency of the initial ringing portion of the click response waveform starts low and increases towards the characteristic frequency (CF) over time (upward frequency glide). In the most apical regions, by contrast, instantaneous frequencies start above CF and decrease with time (downward glide)^4,5^. Mirroring the variation in frequency tuning, response latencies—whether assessed using the wave-front delay of the traveling wave, the mechanical group delay at the wave peak, or the acoustic delay of sounds evoked from the ear—decrease from base to apex when measured in periods of the local CF^5–7^.

Although the frequency dependence of cochlear responses appears well adapted to the analysis and coding of natural sounds^8^, the physical mechanisms responsible for the strategic tonotopic variation in auditory signal processing remain unclear. Until recently, well-controlled measurements of intracochlear motion have been restricted to a handful of locations in the base of the cochlea, and models have therefore focused almost entirely on this region. When combined with the appeal of functionally dissecting the intricate cytoarchitecture of the organ of Corti, the basal bias in the measurements has led to an emphasis on exploring local cochlear micromechanics at the expense of elucidating the role of global variations in cochlear macromechanics and geometry. For example, the width of the basilar membrane and the cross-sectional areas of the cochlear scalae are known to vary in opposite directions^9–13^, but the full functional significance of these opposing tapers has yet to be explained (but see^14,15^). Expanding beyond a narrow focus on the base, a smattering of previous studies have invoked micromechanical^16^ or a mix of micro- and macromechanical processes^17^ to model apical-basal differences in frequency selectivity. However, both the relative contributions of cochlear micro- and macromechanics and whether the same mechanisms can also account for the broad spectrum of apical-basal differences—or for their evident variation across species—remain unknown.

In this paper we apply basic principles of horn acoustics to the tapered geometry of the cochlea. Our analysis explains the observed spatial variation in physiological response properties, including the emergence of a reasonably abrupt apical-basal transition, located midway along the cochlear spiral, across which response characteristics change qualitatively. By design, our account relies entirely on measured anatomical gradients in cochlear macromechanics. Aside from the requisite longitudinal variation in resonant frequency that gives rise to the tonotopic map, the micromechanics of our model organ of Corti are everywhere identical (i.e., the effective admittance of the cochlear partition is assumed almost scaling-symmetric). Furthermore, we demonstrate that the shape of the cochlear duct, in its apt resemblance to an ear horn, enables traveling pressure waves to propagate to their best places with negligible geometric attenuation. The tapered, horn-like geometry of the mammalian cochlea thus plays an essential role both in establishing the apical-basal gradient in cochlear response properties important for auditory signal processing and in enhancing the overall sensitivity of hearing.

## Acoustics of the 3D Cochlea

### Theoretical framework

We assume harmonic time dependence and employ a linearized model of the cochlea. Following linearization, the driving pressure ($P_0$) across the cochlear partition (CP) and the resulting partition velocity ($V_{\mathrm {CP}}$) are related by an effective admittance:

1 $$\begin{aligned} V_{\mathrm {CP}}(x,\omega )= Y_{\mathrm {CP}}(x,\omega )P_0(x,\omega )\;, \end{aligned}$$

where *x* is the distance from the basal end (stapes) and $\omega$ is the angular frequency of the applied stimulus tone.

**Figure 1:**
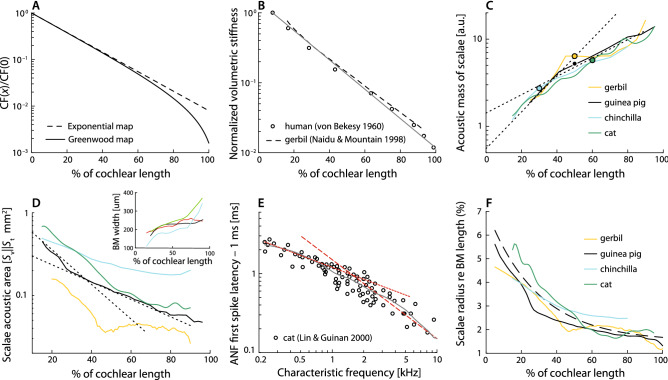
(**A**) Example tonotopic maps for the mammalian cochlea computed using Eq. (2) for the Greenwood function with parameters $\gamma =0.8$ (solid line) and $\gamma =0$ (dashed; purely exponential). In many species, the tonotopic map shows a pronounced downward bend in the apex. (**B**) Longitudinal variation of CP stiffness in human^9^ and gerbil^25^. For comparison, the thin solid line shows the exponential map from panel A. (**C**) Estimated spatial variation of the acoustic mass, $\bar{m}(x)$, of the scalae fluids in four species (solid lines) derived from published morphological data and arbitrarily scaled to emphasize the similarity of their spatial dependence. For comparison, the dotted lines show the exponential curves $e^{x/\ell }$ and $e^{x/2\ell }$ (i.e., the reciprocal of the exponential map in panel A and its square root, respectively). The open symbols indicate the approximate location of the apical-basal transition (${\mathrm {CF}}_{\mathrm {a|b}}$) in each species^7,26^. (**D**) Effective acoustic cross-sectional areas of the scalae [$S_{\mathrm {v}}S_{\mathrm {t}}/(S_{\mathrm {v}}+S_{\mathrm {t}})$] as a function of location in various species. For comparison, the dotted lines show the exponential curves $e^{-x/\ell }$ and $e^{-x/2\ell }$ (i.e., the exponential map in panel A and its square root, respectively). The inset shows the estimated BM widths employed to compute the acoustic mass of the scalae in panel C. (**E**) Wave-front delay in the apex of the cat cochlea, as estimated by subtracting 1 ms from the ANF first-spike latency of the response to acoustic clicks (Fig. 6A of Ref. 27). (Two anomalous data points with delay close to zero are not shown.) The gray line shows the loess trend line, while the dashed and dotted red lines are curves with slopes matching $1/{\mathrm {CF}}(x)$ and $1/{\mathrm {CF}}^{1/2}(x)$, respectively. (**F**) Variation of the radius (*h*) of the scalae along the cochlea in several species, normalized to the cochlea length. The radius was estimated by fitting a circle to the total area of the scalae in all species but the cat, where the radius was estimated as the average of the scala vestibuli and scala tympani height. The dashed line represents the equation ${h(x)}/{L} = 0.0475 e^{-x/2\ell }+0.015$, which captures the overall variation of *h*(*x*) in the different species. Data in panels C,D and F were calculated from data in gerbil^12,28^, in chincilla^29,30^, in guinea pig^10,31^, and in cat^11,32^.

In many mammals, the cochlear frequency-position map is well approximated by the so-called Greenwood function^18^,

2 $$\begin{aligned} {\mathrm {CF}}(x)=A\left( 10^{2.1(1-x/L)}-\gamma \right) \;, \end{aligned}$$

where the species-dependent parameters include the constant *A*, which controls the CF at the base; the length, *L*, of the basilar membrane (BM); and the constant $\gamma$, which determines the location of the low-frequency “bend” where the map transitions from exponential to more linear behavior (Fig. 1A). In multiple species, empirical values of $\gamma$ lie in the range [0.5–1]; in others, such as mouse and guinea pig, the map appears almost exponential ($\gamma \approx 0$)^18–20^. For convenience, we represent the Greenwood function in the form

3 $$\begin{aligned} {\mathrm {CF}}(x)={\mathrm {CF}}(0) e^{-{x \eta (x)}/{l}}\;, \end{aligned}$$

where $l={L}/{2.1\ln (10)}$ and the function $l/\eta (x)$ defines the “local space constant” of the map. In the base of the cochlea, $\eta (x)\approx 1$, meaning that the map is close to exponential. In species with $\gamma >0$, the value of $\eta (x)$ increases as $x\rightarrow L$; the effective space constant therefore decreases in the apex.

The strategy outlined by Duifhuis^21^ yields a tractable description of wave propagation in the 3-D cochlea (detailed in Supplementary Appendixes A,B). We start by introducing the average pressure, $\bar{P}(x)$, defined as the pressure difference between scala vestibuli and scala tympani averaged over the scalae cross-section at location *x*. (For notational simplicity, we henceforth leave the dependence on frequency $\omega$ implicit in most equations.) Mass conservation and Newton’s second law together imply that $\bar{P}(x)$ satisfies a variant of the Webster horn equation familiar from acoustics^22^:

4 $$\begin{aligned} \frac{1}{S} \frac{{d}}{{d}x}\left( {\!S\,\frac{{d}\bar{P}}{{d}x}}\right) +\kappa ^2 \bar{P}= 0\;, \end{aligned}$$

where *S*(*x*) is the effective acoustic cross-sectional area of the scalae [$S(x)=S_{\mathrm {v}}(x)S_{\mathrm {t}}(x)/(S_{\mathrm {v}}(x)+S_{\mathrm {t}}(x))$, with $S_{\mathrm {t}}$ and $S_{\mathrm {v}}$ the areas of scala tympani and vestibuli, respectively] and $\kappa (x)$ is the complex wavenumber. For simplicity, we assume inviscid fluids. The wavenumber then has the value

5 $$\begin{aligned} \kappa (x)=\sqrt{-\alpha \bar{Z}Y_{\mathrm {CP}}}, \end{aligned}$$

where $\bar{Z}(x)=i\omega \rho b/S$, *b* is the width of the BM, $\rho$ is the fluid density, and the function $\alpha (x)=P_0(x)/\bar{P}(x)$ represents the complex ratio of the driving pressure to the average pressure at the same location^21,23^.

Since cochlear wave scattering appears small^24^, we apply the WKB approximation to obtain an expression for the forward-traveling pressure wave:

6 $$\begin{aligned} P_0(x)\approx \alpha (x)\,\bar{P}(0)\, \sqrt{\frac{S(0)}{S(x)}} \sqrt{\frac{\kappa (0)}{\kappa (x)}} \exp {\left( -i\int _0^x\kappa (x')\,dx'\right) }\;. \end{aligned}$$

In the WKB approximation, $\alpha (x)$ becomes^23^

7 $$\begin{aligned} \alpha (x)\approx {\kappa h}/{\tanh (\kappa h)}\;, \end{aligned}$$

where *h*(*x*) is the radius (or height) of the scalae.

Although elegantly compact, the solution here is only formal—the value of $\alpha$ depends nonlinearly on $\kappa$ and vice-versa. Consequently, Eqs. (5) and (7) must be solved by iteration before they can be used to compute $P_0(x)$ (see Supplementary Appendix D)^23^. Nevertheless, the formal solution allows one to elucidate hydrodynamical contributions to the driving pressure. When the wavelengths are long (e.g., at frequencies much lower than the local CF, in the “tail region” of the traveling wave), $|\kappa h|\ll 1$ and $\alpha \approx 1$, so that $P_0$ and $\bar{P}$ are nearly equivalent. Near the peak of the traveling wave, however, the wavelength decreases, becoming small compared to the height of the scalae ($|\kappa h|\gg 1$). In this region, $|\alpha |\gg 1$ and the pressure driving the CP becomes larger than the scalae average. Thus, $\alpha (x)$ can be interpreted as the short-wave pressure gain.

Note that tapering of the cochlear duct affects all aspects of the solution for the pressure [Eq. (6)]. Most obviously, the factor $\sqrt{S(0)/S(x)}$ is absent in box models that ignore this tapering. But variations in scalae height and area also greatly affect both the short-wave pressure gain, $\alpha$ [through Eq. (7)], and the wavenumber, $\kappa$ [through Eq. (5) via $\alpha$ and $\bar{Z}$]. In what follows, we use anatomical and physiological data to determine realistic values for the geometric parameters and then explore their consequences for cochlear function.

## Spatial variation of cochlear acoustic parameters

### Tail-frequency approximation

In the tail region of the traveling wave, the admittance $Y_{\mathrm {CP}}$ appears stiffness-dominated^33,34^, hence

8 $$\begin{aligned} Y_{\mathrm {CP}}(x) \approx {i\omega }/{k(x)}\qquad (\omega \ll 2\pi {\mathrm {CF}}) \;, \end{aligned}$$

where *k*(*x*) is the stiffness of the CP. In this region, the wavelength is long and $\alpha \approx 1$; hence

9 $$\begin{aligned} \kappa (x)\approx \omega \sqrt{\bar{m}(x)/k(x)}\;, \end{aligned}$$

where $\bar{m}(x)=\rho b/S$ is the effective acoustic mass of the fluids.

### Estimates from anatomical data

Figure 1B plots the normalized volumetric stiffness of the CP in humans^9^ and gerbils^25^ as a function of distance from the stapes. In each species, the variation in BM stiffness is well described by

10 $$\begin{aligned} k(x) \sim e^{-{x}/{\ell }}\;, \end{aligned}$$

where $\ell$ is the basal space constant of the cochlear map and the symbol $\sim$ indicates approximate proportionality. (Although $\ell$ depends on species, the two curves appear similar in the figure, where distance is expressed as a percentage of BM length.) We therefore have $k(x) \sim {\mathrm {CF}}(x)$ in the base.

Figure 1C shows values of $\bar{m}(x)$, the acoustic mass of the scalae fluids, calculated from published morphological data in four mammalian species. For comparison, we overlay exponential curves that approximate the spatial variation in the base and apex, respectively. The anatomical data imply that

11 $$\begin{aligned} \bar{m}(x)\sim {\left\{ \begin{array}{ll} e^{x/\ell } &{} \text {in the base}, \\ e^{x/2\ell } &{} \text {in the apex}. \end{array}\right. } \end{aligned}$$

Thus, $\bar{m}(x)$ varies approximately as $1/{\mathrm {CF}}(x)$ in the base and, ignoring possible bends in the map, as $1/\sqrt{{\mathrm {CF}}(x)}$ in the apex. Because the spatial variation of BM width is generally small compared to the variation of the cross-sectional area *S*(*x*) (see the inset in Fig. 1D and note the linear scale), the change in acoustic mass, $\bar{m}(x)$, arises primarily from the acoustic area of the scalae. As illustrated in Fig. 1D, which plots *S*(*x*) in the same four species, the effective area decreases roughly exponentially from base to apex, with slopes somewhere between $-1/\ell$ and $-1/2\ell$, depending on species and location. In particular, in cat, guinea pig, and gerbil, the scalae area varies as $S(x)\sim e^{-x/\ell }$ in the base and $S(x)\sim e^{-x/2\ell }$ in the apex. In the chinchilla, the area is better described by $S(x)\sim e^{-x/2\ell }$ throughout.

### Confirmation from measurements of wave-front delay

The wave-front delay of the traveling wave can be determined directly from the onset delay of the BM click response and/or estimated from the group delay of the BM transfer function at low frequencies. In the model, the wave-front delay expressed in periods of the CF has the value (see Supplementary Appendix B)

12 $$\begin{aligned} \tau _{\mathrm {wf}}\approx {l}\sqrt{{\bar{m}(x)}/{k(x)}}\,{\mathrm {CF}}(x)\;. \end{aligned}$$

In the base of the cochlea, Eqs. (10,11) imply that $\bar{m}/k \sim 1/{\mathrm {CF}}^{2}$, so that $\tau _{\mathrm {wf}}\sim \text {constant}$, independent of *x*. Consistent with this prediction derived from the anatomical data, mechanical measurements in the base confirm that the wave-front delay amounts to a constant number of CF periods, independent of location (see^35^ for a review). Interestingly, this same result can be deduced from measurements of the cochlear input impedance^14^.

In the apex, derivation of the wave-front delay is complicated by the apical bend of the tonotopic map. Nevertheless, simple arguments based on application of Eq. (12) to the anatomical estimates of $\bar{m}(x)$ and *k*(*x*) predict that in the apex, unlike the base, $\tau _{\mathrm {wf}}(x)$ is not constant but decreases with position at least as rapidly as ${\mathrm {CF}}^{1/4}(x)$. Figure 1E shows apical wave-front delays in CF periods estimated from auditory-nerve-fiber (ANF) recordings^27^ as a function of CF. The measurements indicate that $\tau _{\mathrm {wf}}(x)$ varies approximately as ${\mathrm {CF}}^{1/2}$, corroborating this analysis. In guinea pig^6^, ANF estimates of wave-front delay vary as ${\mathrm {CF}}^{0.51}$, in excellent agreement with the trend in cat. A similar dependence is evident in the apex of the chinchilla^5^.

## Implications for Cochlear Signal Processing and Detection

### Efficient wave propagation

**Figure 2:**
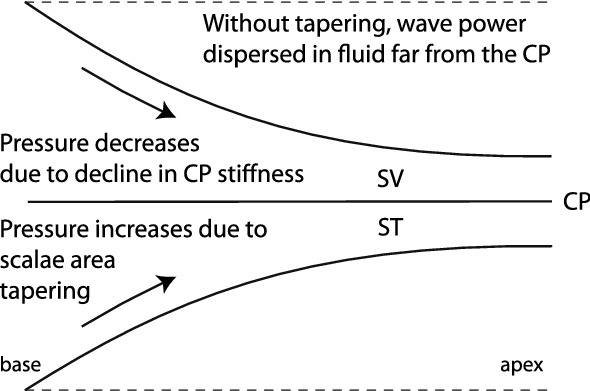
In the tail region of the traveling wave, the horn-like tapering of the cochlear duct boosts the intensity of the traveling wave, compensating for the spatial decay of transpartition pressure that arises from the progressive decline of CP stiffness. In a hypothetical box cochlea (dotted lines), by contrast, the wave power is dispersed throughout the larger fluid volume.

In species such as cat, guinea pig, and gerbil the area of the scalae in the base decreases in parallel with the stiffness of the partition. Consequently, at tail frequencies in the base the prefactor in Eq. (6) for $P_0(x)$ is nearly constant, independent of position. In this regime, where the admittance of the CP is stiffness-dominated, the traveling pressure wave therefore has the form

13 $$\begin{aligned} P_0(x)\approx \bar{P}(0)\,\exp \left( {-i\int _0^x\kappa (x')dx'}\right) \qquad [f\ll {\mathrm {CF}}(x)] \;. \end{aligned}$$

Thus, despite the exponential decline in BM stiffness, pressure waves in the tail region propagate without attenuation—that is, as plane waves of constant amplitude. (Because the wavenumber $\kappa$ is essentially real in this region, the complex exponential term contributes only a phase shift but neither power gains nor losses.) Although the prefactor is not exactly constant in the apex (or in the base of the chinchilla), so that the driving pressure decreases with position, the attenuation space constant ($\sim 8\ell$) is larger than the length of the cochlea. Thus, even in the apex, tail-frequency attenuation with distance is small.

The near-constancy of $|P_0|$ at tail frequencies is a direct consequence of the tapered geometry of the cochlear duct. For example, were the organ of Corti housed within a duct of constant cross section—that is, within a rectangular box, as models often assume—the wave of driving pressure would decay with distance as $e^{-x/2\ell }$, assuming the model parameters were arranged to keep the wavenumbers invariant across models. As in an ear horn, the tapering of the duct boosts the acoustic intensity of the traveling pressure wave. Relative to its value in the tapered geometry, the intensity in the hypothetical box cochlea decays exponentiallly, falling off as $e^{-x/\ell }$ with distance from the stapes. In other words, in the absence of horn-like tapering of the scalae, a significant portion of the power entering the cochlea flows out in the fluids, far from the partition, causing the transpartition driving pressure to decrease with distance as it propagates down along the gradient in BM stiffness. The cartoon in Fig. 2 illustrates the mechanisms behind efficient wave propagation in the tapered cochlea.

In addition to providing a geometric boost to the pressure wave near the partition, the tapered geometry of the scalae has other important benefits for the sensitivity of hearing. In particular, tapering both increases the efficiency of power transmission from the middle ear and reduces the reflection of pressure waves traveling along the cochlea^14^. In the base, at frequencies well below CF, Eqs. (10,11) imply that the characteristic impedance of the wave medium remains real and nearly constant, independent of position:

14 $$\begin{aligned} Z_0(x,\omega ) = \sqrt{\bar{Z}/Y_{\mathrm {CP}}} \approx \sqrt{\bar{m}(x)k(x)} \sim \mathrm {constant}. \end{aligned}$$

With appropriate tapering of the duct, the spatial variation of acoustic mass cancels the variation in stiffness, ensuring reflectionless wave propagation with a constant, resistive $Z_0$.

**Figure 3:**
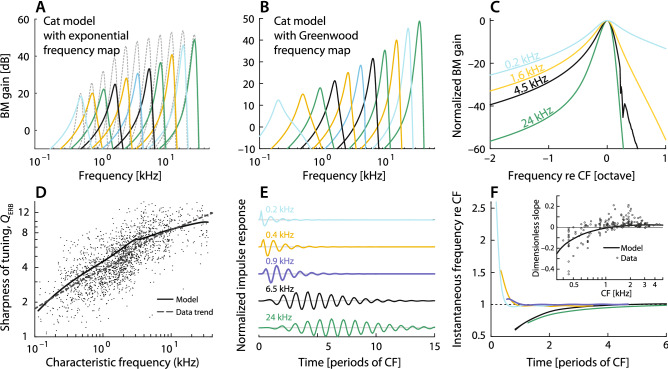
(**A,B**) Model BM gain functions (BM velocity vs frequency re input pressure at the base) at several locations for the model of the cat cochlea when the tonotopic map is assumed either purely exponential (**A**) or of the Greenwood type^36^ (**B**, Eq. (2) with $\gamma =0.8$). Whereas the colored curves in panels (**A**,**B**) represent gain functions calculated in a tapered cochlear model, the grey curves in (**A**) show results for a box model where the cross-sectional area of the duct is assumed constant along the cochlea. (**C**) Gain functions from panel B normalized and plotted versus frequency re CF to emphasize the variation of cochlear tuning along the cochlea. (**D**) Comparison between the model variation in the sharpness of mechanical frequency tuning along the cochlea (measured in $Q_{\mathrm {ERB}}$) and estimates obtained from cat ANF recordings^37^. (**E**) Model BM click responses at multiple CF locations. (**F**) Instantaneous frequencies (normalized to CF) of the click responses from panel E. The inset compares the dimensionless glide slope, defined as the time rate of change of the instantaneous frequency near the peak of the click-response envelope, normalized by the square of the local CF, to values obtained from the cat auditory nerve^4,35^.

### Tonotopic variation of tuning sharpness

As the cross-sectional area of the cochlear duct decreases from base to apex, the height, *h*(*x*), of the scalae tapers correspondingly (Fig. 1F). This taper affects the maximum value of the short-wave pressure gain, $\alpha (x)$, defined as the ratio between the driving and average pressures [approximated by Eq. (7)]. In particular, the value of $\alpha$ near the best place decreases from base to apex (see Supplementary Appendix D). Since the amplitude of the pressure wave reaching the short-wave region varies only slowly with frequency (see previous section), the basal-to-apical decrease in the maximum value of $\alpha (x)$ implies—assuming no strong compensatory mechanism—a corresponding reduction in near-CF BM gain and a concomitant broadening of frequency tuning. To see this, note that as the wave of driving pressure propagates along the cochlea, short-wave hydromechanical pressure gain kicks in as $|\kappa h|$ increases above 1^38^; that is, near the transition from long-wave behavior in the tail to short-wave behavior in the tip. Although quantitative analysis is complicated by the nonlinear equations for $\alpha$, the key result can be understood qualitatively by imaging a family of models with identical wavenumbers but different scalae heights, *h*. At any given frequency, waves traveling in models with relatively large values of *h* near the best place (such as those representing waves that peak in the base of the cochlea) become short-wave sooner and therefore have greater short-wave gain and sharper tuning. By contrast, waves in models (or regions of the cochlea) with smaller values of *h* (i.e., the apex) remain long-wave longer and so accumulate less short-wave gain at the peak (broader tuning).

We elucidate this phenomenon further using an active model of the CP admittance (see Supplementary Appendix C; see also Supplementary Appendix G for the effects of active amplification on cochlear hydrodynamics)^38,39^. For purposes of illustration, and in order to highlight the role of macromechanical processes, we stipulate that the sharpness of “micromechanical” tuning in the model be everywhere identical. For ease of comparison with the experimental data, model parameters are tailored to the cat. However, because apical/basal differences in both anatomy and physiology are qualitatively similar across mammalian species (see^7^ and our Fig. 1) our general findings about their geometric origins also apply to other species. Inter-species differences arise through factors such as variations in the tonotopic map and the location of the apical/basal transition (Fig. 1C). As an example of model generality, Supplementary Appendix E shows results for a model tailored to the gerbil.

Figure 3A shows model BM gain functions computed at different cochlear locations, assuming a purely exponential tonotopic map. In accordance with our deductions, both the gain at CF, and tuning sharpness decrease from base to apex. Furthermore, the slope of the high-frequency cut-off, very steep in the base (${\mathrm {CF}}>3\,$kHz), becomes shallower in the apex. This model trend resembles that seen in neural data and is a consequence of correlations between cut-off slope and wave-front delay predicted by simple considerations of cochlear macromechanics (Supplementary Appendix B). For comparison, the dotted gray curves in Fig. 3A plot the BM gain functions for a model with the same parameters except that the radius (and thus the cross-sectional area) of the scalae is held constant (box model). Although the pressure decay inherent in box models (see previous section) decreases the peak BM gain with distance from the stapes, the shapes of the gain functions appear nearly scaling-symmetric throughout the cochlea.

Cochlear frequency selectivity is controlled, in part, by the form of the tonotopic map. The apical bend evident in the maps of multiple species (Fig. 1A) has the effect of warping the frequency axis of Fig. 3A at its low-frequency end. Figure 3B shows model BM transfer functions, computed at the same locations used in Fig. 3A, when the cochlear map is taken to match the non-exponential form measured in the cat [$\gamma =0.8$ in Eq. (2)]^36^. In this case, transfer functions at the lowest CFs resemble nonlinearly “stretched” versions of those obtained using the exponential map (Fig. 3A). This frequency-warping effect produces apical transfer functions that appear “flipped” relative to their basal cousins: in the extreme apex, low-frequency slopes are steeper than high-frequency flanks (Fig.3C). This asymmetry between apical and basal frequency responses is found in ANF tuning curves from cat and chinchilla^1,3,40^. Figure 3D shows the quality factor of the model’s transfer function, measured as the equivalent rectangular bandwidth ($Q_{\mathrm {ERB}}$), along with the $Q_{\mathrm {ERB}}$ estimated from cat ANF tuning curves^37^.

### Downward glides in the apex

The qualitative differences in the shapes of apical and basal BM transfer functions (Fig. 3B) have significant effects on their respective time-domain responses to acoustic clicks. Figure 3E shows model BM click responses at different cochlear locations. In the basal and middle turns of the cochlea, response onsets are dominated by low-frequency components; the result is an upward glide in which the instantaneous frequency of the oscillation increases over time, approaching CF from below (see Fig. 3F). At CFs below about 1 kHz, by contrast, response onsets are dominated by frequency components greater than CF, producing a downward glide. (For CFs near 1 kHz, the glide duration is very short.) The inset in Fig. 3F shows that the model captures the observed sign, magnitude, and CF-dependence of the dimensionless glide slope. Never previously accounted for by models of cochlear mechanics, the emergence of frequency glides of different directionalities in the base and apex has been a striking, unexplained feature of ANF recordings in cat and chinchilla^4,5^.

### Otoacoustic delays and tuning ratios

Neural and otoacoustic measurements reveal strong correlations between the sharpness of ANF frequency tuning ($Q_{\mathrm {ERB}}$) and the latency ($N_{\mathrm {SFOAE}}$, in periods) of stimulus-frequency OAEs (SFOAEs) at frequencies near CF^7^. Under the assumption that SFOAEs arise predominantly from a region near the peak of the traveling wave, these correlations find a natural explanation in the inverse relationship between bandwidth and delay expected in resonant systems. Defined as the ratio of the trend lines for each variable ($Q_{\mathrm {ERB}}/N_{\mathrm {SFOAE}}$), the tuning ratio remains fairly constant in the base of the cochlea, but gradually increases severalfold below the apical-basal transition (${\mathrm {CF}}<\mathrm {CF}_{\mathrm {a|b}}$)^7^. This curious, apical increase in the tuning ratio appears to be a near-universal feature of the mammalian cochlea; it implies that low-frequency SFOAE delays are substantially shorter than expected based on the change in sharpness of tuning along the cochlea.

**Figure 4:**
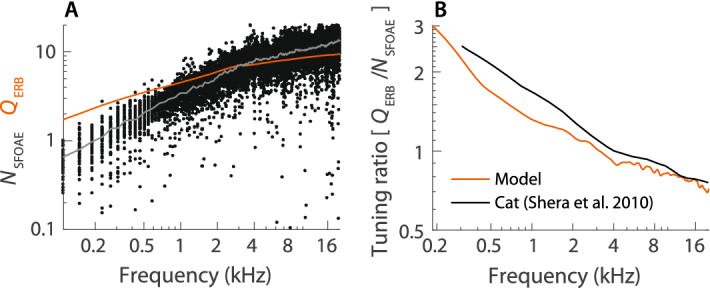
(**A**) Model SFOAE delays $N_{\mathrm {SFOAE}}$. Dots indicate individual simulations and the gray line represents their median at each frequency. For comparison, the red line plots $Q_{\mathrm {ERB}}$ from Fig. 3D at the corresponding CFs. SFOAEs were simulated in 128 different “ears” by introducing small random irregularities in the partition admittance [methods detailed in^23^]. (**B**) Comparison between the model tuning ratio ($Q_{\mathrm {ERB}}/N_{\mathrm {SFOAE}}$) and that obtained from otoacoustic and neural data in cat (Fig. 9 of Ref. 7).

Our simple model reproduces this result and provides an explanation. Conceptually, the delay $N_{\mathrm {SFOAE}}$ can be decomposed into two components: (i) a delay, fairly independent of $Q_{\mathrm {ERB}}$, associated with round-trip propagation of the pressure wave between the middle ear and the peak region and (ii) a delay, roughly proportional to $Q_{\mathrm {ERB}}$, associated with the build-up of pressure within the peak region. In this simplified view, our analysis indicates that although component (ii) decreases in parallel with $Q_{\mathrm {ERB}}$ from base to apex, component (i) decreases faster in the apex than the base, independent of $Q_{\mathrm {ERB}}$ (see Fig. 1E). Figure 4A compares model values of $N_{\mathrm {SFOAE}}$ and $Q_{\mathrm {ERB}}$, showing that $N_{\mathrm {SFOAE}}$ decreases faster than $Q_{\mathrm {ERB}}$ at low frequencies. Figure 4B compares the resulting tuning ratios with those estimated in the cat^7^; the agreement is compelling, especially considering the simplicity of the model and its assumptions.

## Discussion

We demonstrate that a simple model incorporating realistic tapering of the cochlear duct and basilar membrane can account for many of the most salient differences between responses, both mechanical and neural, measured in the apical and basal regions of the cochlea. By purposefully eliminating possible deviations from scaling in cochlear micromechanics, our analysis shows that gradients in hair-cell properties or organ-of-Corti cytoarchitecture, although surely present, appear unnecessary to explain the observed responses. Furthermore, the relative simplicity of the model clearly exposes the underlying physical mechanisms, emphasizing their analogy to basic principles of horn acoustics. Importantly, our analysis demonstrates that the tapered, horn-like geometry of the cochlea not only subserves important apical-basal variations in cochlear signal processing, but also helps boost the sensitivity of hearing, especially at low frequencies. Tapering not only ensures efficient transmission of sound energy through the middle ear to the cochlea^14^, it allows the wave of driving pressure to propagate at tail frequencies as a plane wave of constant amplitude—despite the rapid spatial variation of BM stiffness, the wave travels without significant reflection or attenuation. Realizing these multiple benefits for cochlear signal processing requires an apparent coordination among diverse geometric and functional parameters. At tail frequencies, the coordination results in the elimination of significant spatial variations in both the magnitude of the driving pressure [i.e., the prefactor in Eq. (6) for $P_0(x)$] and in the characteristic impedance of the wave medium. We refer to the design principle that underlies this remarkable coordination as “tapering symmetry”^14^.

By preventing the spatial decay of traveling pressure waves in the long-wave region, tapering reduces the demand for their subsequent amplification by active mechanisms in the short-wave region near the wave peak. Note that by shortening the height of the scalae in the apex, tapering decreases the potential boost provided by the short-wave pressure-gain factor ($\alpha$) in that region (see Eq. (7) and Fig. 3A,B). One can therefore imagine an alternative cochlear design: a box model of constant height constructed to yield apical peak wave amplitudes similar to those obtained in the more realistic geometry. After necessarily attenuating pressure waves as they pass through the base, the box cochlea—with its relatively larger values of $|\alpha |$ in the apex, supplemented as necessary with stronger outer hair cell-based amplification—could provide these attenuated waves a compensatory boost close to their best place. This strategy of reamplifying the signal after initially allowing it to decay suffers from the problem that the reamplification process boosts not only the signal but also the internal noise, rendering signal detection more difficult. The issue is especially acute at low frequencies, where the amplitudes of biological and thermal noise typically increase. Evolution appears to have stumbled upon the principle that, rather than trying to compensate for past mistakes—here, by first attenuating and then reamplifying—it often proves better to avoid those mistakes at the outset.

The apical reduction in short-wave gain caused by tapering naturally results in reduced frequency selectivity in the apex. Although our basal biases associate broader tuning with impairment, the maintenance of sharp tuning at low frequencies may provide little functional advantage for hearing. Indeed, if cochlear responses themselves are any guide, sharp low-frequency tuning may well be detrimental for auditory signal processing. Because the inner hair cells and auditory nerve can resolve and phase-lock to the temporal fine structure of sound components reaching the apex^41^, the broad tuning and shallow high-frequency slopes found in the apex (see Fig. 2C) imply that low-frequency stimuli excite relatively large portions of the cochlea and consequently recruit a large number of auditory-nerve fibers. The notion that the auditory system benefits from broader tuning and reduced local cochlear gain in the apex appears consistent with studies that report significant advantages for the coding of natural sounds^8^.

Sasmal and Grosh^17^ recently presented a finite-element model of the guinea-pig cochlea, concluding that three principal factors underlie the apical-basal variation of tuning in the model: (i) the tapering of cochlear dimensions, whose physical effects we elucidate here; (ii) fluid viscosity; and (iii) a micromechanical change in the orientation of the outer hair cells (OHCs) within the organ of Corti such that they provide reduced BM amplification in the apex compared to the base. In their model, the principal effect of viscosity is to eliminate spurious peaks and notches caused by wave reflection from the helicotrema [see also^15^]. Although the simplified treatment presented here ignores the helicotrema boundary, numerical calculations that include viscosity agree well with their finding. A key difference between the responses predicted by our model and that of Sasmal and Grosh is that the mix of micro- and macromechanical processes in their model produces very broad mechanical tuning in the apex, necessitating the inclusion of a 6-dB/octave highpass filter—hypothesized to represent the putative effects of adaptation of the IHC mechanoelectric-transduction (MET) current—between mechanical and neural tuning. Although the role of MET adaptation in mammalian hair cells remains controversial^42,43^, it appears unlikely that IHC MET adaptation in vivo manifests as a large current decay^43,44^ capable of introducing significant frequency dependencies in IHC responses (see also^45^).

Although classic measurements indicate that mechanical and neural tuning are similar in the apex of the guinea pig and chinchilla^2,46^, more recent measurements from the guinea pig^47^ suggest that mechanical tuning in the apex is fundamentally low-pass in character. If so, accounting for the tuning of low-frequency ANFs would therefore require the interposition of a so-called “second filter,” such as the adaptation mechanism discussed above^17^. However, these recent mechanical measurements were obtained not from the BM, but from structures located close to the reticular lamina, near the top of the OHCs—that is, from a region within the organ of Corti whose mechanical responses are more broadly tuned than those of the BM and auditory nerve, at least in mouse and gerbil^48,49^. In this regard, the present model predicts that the tuning of OHC forces relative to BM motion is broader in the apex than in the base (Supplementary Appendix F). Insofar as the internal motion of the organ of Corti is dominated by deformations due to OHC somatic motility^50^, the model thus accounts for the discrepancy between the classic data and more recent measurements. Although mechanical and neural tuning are surely not everywhere identical, our results support the idea that ANF tuning largely reflects the overall transverse motion of the CP throughout the cochlea, implying that no special second filter is needed to explain the data.

Our simplified model does not, of course, preclude the existence of important, local micromechanical specializations that vary along the cochlear spiral. Furthermore, although the motion of the BM appears to closely mirror the slow-traveling pressure wave in the base of the cochlea^34^, important deviations may arise in the apical turn, where the BM is much more compliant (Fig. 1B) and its motion may be influenced by deformations of the organ of Corti that are not strongly coupled with the transpartition pressure wave^51^. Nevertheless, our analysis highlights the key role played by the global, macromechanical geometry and hydrodynamics of the cochlea. By shaping the wave of driving pressure, the cochlea’s horn-like acoustics gives rise to many of its most puzzling, and functionally significant, response characteristics. These include systematic spatial variations in the sharpness of tuning, the frequency-dependent delay of otoacoustic emissions, and the peculiar patterns of dispersion (i.e., glides) observed in the responses of the auditory nerve.

## Supplementary information

Supplementary Information.

## Data Availability

The code generated in this study is available at https://www.mechanicsofhearing.org/apg.
