# Supplementary material for: The cochlear ear horn: geometric origin of tonotopic variations in auditory signal processing
Source: Sci Rep. 2020 Nov 25;10:20528. doi: 10.1038/s41598-020-77042-w (PMC7689495; doi:10.1038/s41598-020-77042-w)
Supplement: Supplementary file 1 — Supplementary Information. [file 41598_2020_77042_MOESM1_ESM.pdf]

The cochlear ear horn: Geometric origin of tonotopic variations in auditory signal processing

Alessandro Altoè*and Christopher A. Shera†

Auditory Research Center, Caruso Department of Otolaryngology,
University of Southern California, Los Angeles, CA

Supplementary Appendices A–G

Appendix A

Hydrodynamics of the 3-D cochlea

For simplicity, we assume that the cross-section of the scalae is a rectangle of width w and height h (Fig. S1). Nevertheless, the approximations, derivations, and results shown here also apply to models where the shape of the cochlear cross-section is different (e.g., circular). We consider a wave elicited by a tone of angular frequency ω; to simplify the notation, the dependence on ω is generally omitted. The flexible part of the cochlear partition (CP) spans a fraction of the cochlea width, and we make no assumption about whether the CP is centered in the radial direction.

Laplace's equation for the pressure in the fluid is

$$\frac{\partial^2 p}{\partial x^2} + \frac{\partial^2 p}{\partial y^2} + \frac{\partial^2 p}{\partial z^2} = 0 \; . \qquad \text{(S.1)}$$

Employing a separation of variables, the Laplace equation yields

$$\kappa_x^2 + \kappa_y^2 + \kappa_z^2 = 0 \; , \qquad \text{(S.2)}$$

where $\kappa_{x,y,z}$ indicates the local wavenumber in the corresponding direction. The above equation states that the pressure at any point in the cochlea can be determined as a superposition of modes determined by the boundary conditions:

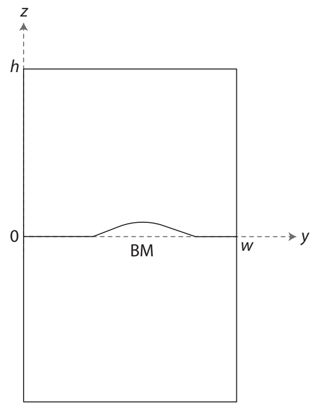

Figure S1: Cross section of a rectangular 3D model of the cochlea.

$$\begin{cases} \dfrac{\partial p}{\partial y} = 0 & z = h, \\ \dfrac{\partial p}{\partial z} = 0 & y = 0, w \; . \end{cases} \qquad \text{(S.3)}$$

Given κ_x, the amplitude of each mode depends on the profile of the transverse motion of the CP in the radial (y) direction. Neglecting compression waves traveling at the speed of sound in water the solution for the pressure is largely dominated by the mode defined by $\kappa_y = 0$ because the width of the CP is on the order of 100 µm and the transverse displacement produced by low-level sounds is on the order of few nm. With these simplifications, one obtains

$$\kappa_z^2 = -\kappa_x^2 \; . \qquad \text{(S.4)}$$

*altoe@usc.edu

†also at Department of Physics & Astronomy, University of Southern California, Los Angeles, CA; christopher.shera@usc.edu

In the limit that the space constant characterizing the tapering of the scalae height is larger than the height of the scalae, we can employ the averaging procedure detailed by Duifhuis [4] for 2D box models.

Following Duifhuis and introducing the scalae-averaged pressure, $\bar{P}(x)$, one can apply Newton's second law and mass conservation to obtain

$$\frac{d\bar{P}}{dx} = -\, i\omega \frac{\rho}{S(x)} U(x), \tag{S.5}$$

and

$$\begin{aligned} \frac{dU}{dx} &= -\, b(x) V_{\rm BM}(x) & \text{(S.6)} \\ &= -\, \alpha(x) b(x) Y_{\rm CP}(x) \bar{P}(x) \;, & \text{(S.7)} \end{aligned}$$

where the fluids are assumed inviscid, ρ is the fluid density, $S(x)$ is the effective acoustic cross-sectional area of the scalae, $b(x)$ the width of the CP, $V_{\rm BM} = Y_{\rm CP} P_0$, and $P_0 = \alpha \bar{P}$. Uncoupling these equations yields

$$\frac{1}{S}\frac{d}{dx}\left(S\,\frac{d\bar{P}}{dx}\right) + \kappa^2 \bar{P} = 0 \;, \tag{S.8}$$

where $\kappa(x)$ is the complex wavenumber ($\kappa^2 = -\alpha \bar{Z} Y_{\rm CP}$) with

$$\bar{Z}(x) = i\omega\rho b(x)/S(x) \;. \tag{S.9}$$

Equation (S.8) can be simplified by introducing the change of variables defined by

$$\chi(x) = S_0 \int_0^x \frac{dx'}{S(x')} \;, \tag{S.10}$$

where $S_0 = S(0)$. The new spatial variable χ represents the effective "acoustic distance" from the stapes [18]—it increases more rapidly with x when the scalae areas are small and reduces to the conventional distance x when the scalae areas are constant. All dependent variables are now regarded as functions of χ. With this change, Eq. (S.8) becomes

$$\frac{d^2\bar{P}}{d\chi^2} + \hat{\kappa}^2 \bar{P} = 0 \;, \tag{S.11}$$

where $\hat{\kappa} = (S/S_0)\kappa$ is the wavenumber in the χ domain. Applying the WKB approximation yields

$$\bar{P}(\chi) \approx \bar{P}(0)\sqrt{\frac{\hat{\kappa}(0)}{\hat{\kappa}(x)}}\exp\left(-i\int_0^\chi \hat{\kappa}(\chi')\,d\chi'\right) \;. \tag{S.12}$$

Converting this solution back into the x domain yields

$$P_0(x) \approx \alpha(x)\,\bar{P}(0)\sqrt{\frac{S(0)}{S(x)}}\sqrt{\frac{\kappa(0)}{\kappa(x)}} \;\times \; \exp\left(-i\int_0^x \kappa(x')\,dx'\right) \;, \tag{S.13}$$

as given in the main text.

Appendix B

Wavefront delay and high-frequency cut-off

For what follows, it proves convenient to rewrite the equations in terms of frequency normalized to the local CF using the variable $\beta(x,f) = f/\mathrm{CF}(x)$. Using the tonotopic map, this becomes

$$\beta(x) = \beta_0 e^{x\eta(x)/l} \tag{S.14}$$

so that

$$\frac{d\beta}{dx} = \left[\eta(x) + x\frac{d\eta}{dx}\right]\frac{\beta}{l} \;. \tag{S.15}$$

In the base of the cochlea, where simple quantitative statements about the relationship between wavefront delay and cochlear dimensions can be made, the approximations $\eta(x) \approx 1$ and $d\beta/dx \approx \beta/\ell$ work well. In the apex, although the equations can be solved, the low frequency "bend" of the cochlear map complicates the solutions to the point that there is no advantage to showing those over purely numerical results.

To determine the wave-front delay and its relation to cut-off, it suffices to ignore the prefactor in Eq. (S.13) and focus on the complex exponential. Defining κ_β as the wavenumber in the β domain,

$$\kappa_\beta = \frac{dx}{d\beta}\kappa \;, \tag{S.16}$$

enables one to rewrite the complex exponential ($e^{i\phi}$) as

$$\phi(\beta) = -\int_{\beta_0}^{\beta} \kappa_\beta \, d\beta' \;. \tag{S.17}$$

At tail frequencies, where κ_β is real, the group delay in periods of the CF is given by

$$\tau_{\rm wf} = -\frac{1}{2\pi}\frac{d\phi}{d\beta} = \frac{\kappa_\beta}{2\pi} \ . \tag{S.18}$$

The tail-frequency approximation to the wavenumber discussed in the main text [Eq. (9)] implies that

$$\kappa_\beta \approx \omega\frac{dx}{d\beta}\sqrt{\frac{\bar{m}(x)}{k(x)}} \ , \tag{S.19}$$

so that

$$\tau_{\rm wf} = l\sqrt{\frac{\bar{m}(x)}{k(x)}}{\rm CF}(x) \ , \tag{S.20}$$

as given in the main text.

Far enough from the helicotrema, the onset of the CP click response is dominated by components well below CF, and hence $\tau_{\rm wf}$ coincides with the onset latency of the response (i.e., with the wavefront delay of the traveling wave). As one approaches the helicotrema in the apex, both $\bar{m}(x)/k(x)$ and $dx/d\beta$ decrease, and therefore the wavefront delay also decreases. For an exponential frequency map, given the constraints on $\bar{m}$ and k deduced from the anatomical data, one obtains $\tau_{\rm wf} \propto \Omega(\omega_{\rm c}^{1/4})$, where Ω indicates asymptotic growth at least as fast as its argument. As frequency decreases, the low-frequency bend of the tonotopic map produces a steeper decline in the value of $\tau_{\rm wf}$.

When the wavenumber κ_β has a significant negative imaginary part, the complex exponential $e^{-i\int\kappa_\beta d\beta'}$ dominates the behavior of the high-frequency cut-off observed in mechanical and neural data. For simplicity, we assume that $\alpha \approx 1$ (i.e., the long-wave approximation); this approximation appears not unsatisfactory in the apex, where the scalae radius and the short-wave gain factor are small. We can then write the wavenumber in the form

$$\kappa_\beta = \frac{dx}{d\beta}\kappa = 2\pi\tau_{\rm wf}\sqrt{\hat{Y}_{\rm CP}}, \tag{S.21}$$

where $\hat{Y}_{\rm CP} = [k(x)/i\omega]Y_{\rm CP}$ so that $\hat{Y}_{\rm CP} \approx 1$ in the tail. The cut-off occurs because $\hat{Y}_{\rm CP}$ has a positive imaginary part above CF (i.e., the mechanics of the CP contain a significant dissipative component). The complex exponential in the pressure becomes

$$e^{-i\int\kappa_\beta d\beta'} = e^{i2\pi\tau_{\rm wf}\int\sqrt{\hat{Y}_{\rm CP}}d\beta'}, \tag{S.22}$$

indicating an exponential decay of pressure beyond the peak. Equation (S.22) implies that the larger the wave-front delay, $\tau_{\rm wf}$, the more steeply the pressure decreases above CF; conversely, the smaller $\tau_{\rm wf}$, the gentler the cut-off.

Appendix C

Model of the CP admittance

To a first approximation, we assume that the center of mass of the cochlear partition moves in proportion to the BM. We therefore employ the BM admittance, first deduced by Zweig [24], that we have previously shown provides an excellent fit to BM transfer functions measured in the base of the gerbil and in the apex of the mouse cochlea [1] . In particular, we assume that

$$Y_{\rm CP}(x,\omega) = \frac{i\omega(1+i\omega\tau/\omega_{\rm c})}{m_a(x)(-\omega^2+2i\omega\zeta\omega_{\rm c}(x)+\omega_{\rm c}^2(x))} \ , \tag{S.23}$$

where τ is the strength of the active force, m_a is the acoustic mass (or mass per unit area) of the BM (see [1]), and ζ is the damping factor. Rewriting this in terms of the scaling variable $\beta = \omega/\omega_{\rm c}$ yields

$$Y_{\rm CP}(\beta) = \frac{i\omega}{k(x)}\frac{1+i\beta\tau}{1-\beta^2+2i\zeta\beta} \ , \tag{S.24}$$

where the CP stiffness $k = m_a\omega_{\rm c}^2$. Imposing the constraint that τ and ζ be constant ensures that the local micromechanics, as reflected in the ratio between BM velocity and driving pressure, retain the same sharpness of tuning along the cochlea. The parameters for cat are $\tau = 1.3$ and $\zeta = 0.12$. Consistent with our analysis, we assume that partition stiffness (k) and fluid acoustic mass ($\bar{m}$) vary with position as deduced from the experimental data, with the apical-basal transition at ${\rm CF}_{\rm a|b} \approx 3\,$kHz [20].

Appendix D

Numerical calculation of the short-wave gain factor

Having defined the geometry of the cochlea and the admittance of the partition, we can numerically compute the model's responses. Unfortunately, this is not as straight-forward as it appears, because the solution for $P_0(x)$ given by Eq. (5) is only formal. Note that $P_0(x)$ depends on $\alpha(x)$—both directly via the prefactor and indirectly via the wavenumber, since κ depends on α [see Eq. (6)]—but that $\alpha(x)$, in turn, depends on $P_0(x)$ via the definition $\alpha = P_0/\bar{P}$. Thus, the solution must be obtained by iteration [19].

To determine the value of $\alpha(x)$, we adopt the WKB approximation to $\alpha(x)$ given by Eq. (6) and solve by iteration the following pair of equations:

$$\begin{aligned} \kappa &= \sqrt{-\alpha \bar{Z} Y_{\mathrm{CP}}}\ , \\ \alpha &= \frac{\kappa h}{\tanh(\kappa h)}\ . \end{aligned} \tag{S.25}$$

At frequencies below the local CF, the iterative numerical solution obtained by starting from the long-wave approximation rapidly converges. Above CF, however, where Y_{CP} is dominated by dissipative forces [14], solution is complicated by the existence of multiple solutions and the iteration can fail to converge. Physically, the multiple solutions represent evanescent modes (e.g., standing waves in the z direction) and are responsible for the sharp peaks and notches often observed in BM transfer functions at frequencies above CF (see [22, 24]). In addition, the WKB approximation to α we employ [Eq. (6)] assumes a single mode and therefore breaks down well above CF (see [23]). We largely circumvent these problems in our calculations by forcing the iteration to converge on the value of κ whose real and imaginary parts have the same sign as the wavenumber obtained using the long-wave approximation, which neglects evanescent modes. Figure S2A,B show values of $\alpha(x)$ for waves of different frequencies as computed in the model used to obtain the transfer functions in Fig. 3B. Figure S2C,D show the corresponding real and imaginary parts of the wavenumber (κ).

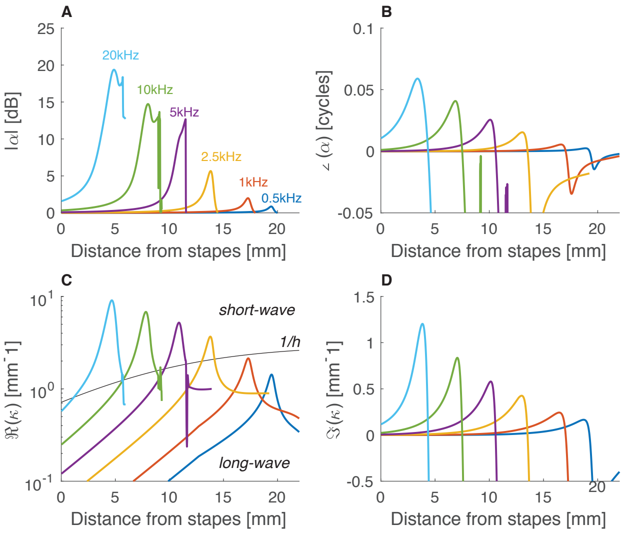

Figure S2: (**A,B**) Short-wave pressure gain factors $\alpha(x)$ along the cochlea computed by iterating Eqs. (S.25) for waves of different frequencies (color coded in A). Panels A and B give the magnitude and phase, respectively. (**C,D**) Real and imaginary parts of the wavenumber (κ). The thin line in panel C shows the value of $1/h(x)$ along the cochlea: when the wavenumber is smaller than this value, wavelengths are larger than the scala height, and the long-wave approximation is expected to hold well. When the wavenumber is larger than $1/h(x)$, the long-wave approximation breaks down and the pressure driving the motion of the CP undergoes an effective boost due to the short-wave hydrodynamics (see panel A and [24, 1]). Note that in the long-wave region, the curves in panel C are approximately parallel, reflecting the approximate scaling behavior of the wavenumber at tail frequencies. Note also that at apical locations the pressure wave remains effectively long-wave, never entering a short-wave region. For visual clarity, the values of α and κ are shown only at locations where the magnitude of the BM transfer function is within 80 dB of its peak value.

Appendix E

Results in a gerbil model

Because of the wealth of information about apical-basal differences in the cat [e.g., 6, 8, 2, 17], we presented the results of a model tailored to the this species in the main text. We show here that analogous results are obtained in a model tailored to the gerbil cochlea, where a comparison with mechanical data is possible. For the ger-

bil, we assume a Greenwood-type tonotopic map with $\gamma = 0.6$ [5, 9] and a total cochlear length of 12.5 mm. The model parameters were adjusted slightly from [1] ($\tau_{\rm wf} = 1.1$, $\tau = 1.3$ and $\zeta = 01.3$) in order to yield a better match to measurements of the sharpness of tuning obtained from auditory-nerve fibers (ANFs) in the base of the cochlea. As a result, the model transfer function shown in Fig. S3A,B is somewhat more sharply tuned than the BM transfer function measured at 40 dB SPL. We are not too concerned about this discrepancy, both because the difference between model prediction and data (roughly 4 dB at the peak) is comparable to those observed between animals of the same species (see Fig. 3B of [1]) and because the experimental data were obtained at sound levels likely to be in the compressive regime [21], where tuning is broader than at sound levels near ANF threshold. Figure S3C shows that the model provides a good match to the sharpness of tuning measured in the auditory nerve [15].

For completeness, Figs. S3D,E show the magnitude and phase of a BM transfer function obtained near the apical end of the gerbil model (CF $\approx$ 1 kHz). The shape of the transfer function magnitude in Fig. S3D resembles those obtained in the apex of the cat model (see Fig.3B in main text and Fig. S5).

Appendix F

Active forces in the base and in the apex

The active force term in the model of the CP partition is

$$f_{\rm act} = i\beta\tau P_0. \qquad \text{(S.26)}$$

This mathematical term provides only a phenomenological description of the effect of the active cochlear process on the response of the BM, but its mathematical form is firmly based on the experimental data [24, 1]. We have previously shown that this active term provides a compelling phenomenological description of OHC forces, yielding nonlinear model responses that are strikingly similar to mechanical responses measured near the reticular lamina (top of the

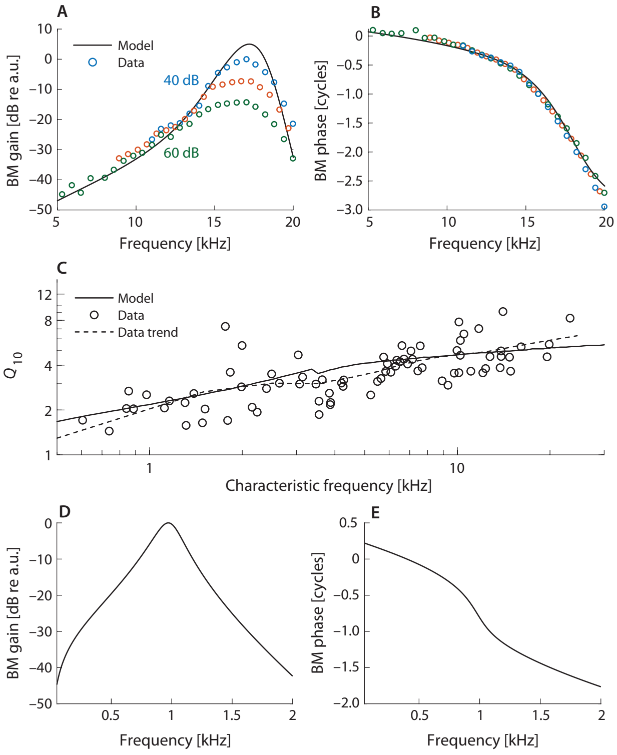

Figure S3: (**A,B**) Comparison between model and measured BM transfer functions in the base of the gerbil cochlea. Because the model parameters were chosen to match the sharpness of tuning estimated from auditory-nerve recordings near threshold, the model transfer function is more sharply tuned than the reference data. Data courtesy of Wei Dong, Loma Linda University. (**C**) Comparison between the predicted variation in the sharpness of mechanical frequency tuning along the cochlea, as quantified by the Q_{10} (quality factor at 10 dB below the peak), and estimates obtained from gerbil ANF recordings (data from [15]). (**D,E**) Predicted BM transfer function in the apical turn of the gerbil cochlea (CF $\approx$ 1 kHz).

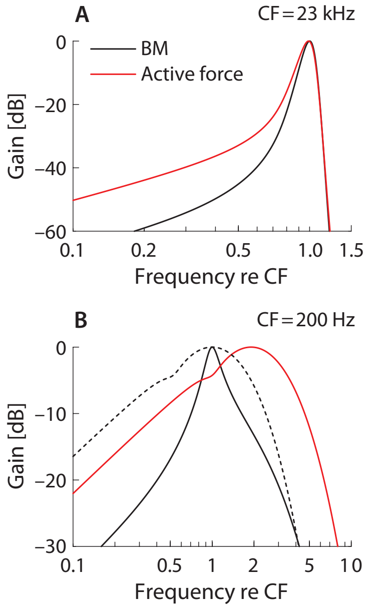

Figure S4: Comparison of the frequency tuning of the BM (black lines) and that of the model's active force (red line) in the base (**A**) and apex (**B**) of the cochlea. The dashed line in panel B shows the frequency response of the active force on an axis normalized by the its own best frequency rather than by the BM CF.

OHCs) in the 9–10 kHz region of the mouse cochlea [1].

Although the local mechanics of the partition are described by the same equation throughout the cochlea, the manifestation of the active force in the model appears qualitatively different in the base and in the apex (Fig. S4). This difference arises because of apical-basal differences in the frequency tuning of the transpartition pressure resulting from the global geometry of the cochlea.

In both the apex and the base, the active force is more broadly tuned than the BM. At basal locations (Fig. S4A), the differences in tuning between the active force and the BM appear qualitatively similar to those observed between the reticular lamina (RL) and BM motion [11, 7, 3]. In the apex (Fig. S4B), the active force is much more broadly tuned and peaks at a frequency higher than the BM. For a visual comparison of tuning, the dashed line in Fig. S4B shows the frequency response of the active force on an axis normalized to its best frequency. That is, our model suggests that the broad tuning of the internal organ of Corti motion measured in the apex of the guinea pig [10]—and the apparent mismatch between neural and mechanical tuning—results from the broad tuning of the OHC forces rather than the broad tuning of the overall transverse motion of the partition. The truth of this model-based conjecture remains an interesting empirical question.

Appendix G

Effects of the active process on cochlear hydrodynamics

As a traveling wave approaches its best place, the cochlear amplifier acts not only to increase its amplitude but also to decrease its group velocity, thereby increasing the group delay of the response [e.g., 13]. This coordinated action is a consequence of causality, as manifest through the Kramers-Krönig relations (see [16]). Physically, this means that the active process modifies both the real and imaginary parts of the wavenumber near the best place, effectively exploiting cochlear hydrodynamics to boost the the transpartition pressure wave via spatially distributed amplification [1].

Equation (S.25) implies that the wavenumber, κ, and the short-wave pressure gain factor, α, are intimately related. Both depend strongly on the radius of the scalae, h. As a result, our analysis reveals that the global, tapered geometry of the cochlea introduces a significant spatial dependence on traveling-wave amplification (Fig. S2A,D). As we demonstrate in the model, this conclusion holds even when the local micromechanical processes that regulate the activity of the cochlear amplifier are perfectly scaling symmetric. Furthermore, the result does not depend on the particular model form of the CP admittance—all active cochlear models necessarily include processes that modify both the real and imaginary parts of the wavenumber (see [16]).

Figure S5 illustrates additional important effects of the global hydrodynamics. The figure compares the magnitude and phase of the BM transfer function in the active cat model

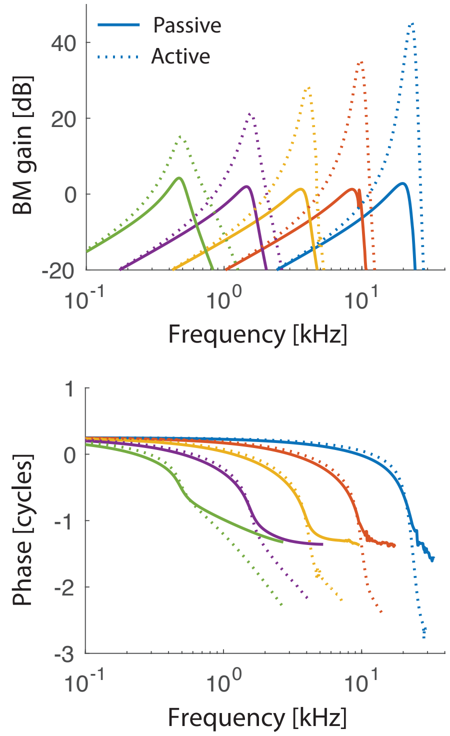

Figure S5: Comparison of BM transfer functions computed in active and passive versions of the model of the tapered cat cochlea.

of the tapered cochlea, with those obtained in the corresponding passive model (obtained by setting $\tau = 0$ in Eq. S.23). Interestingly, the model predicts that the active process causes the *in vivo* near-CF gain relative to that measured *post mortem*, to decrease with distance from the stapes. These model results, which are consistent with both classic and more recent experimental recordings from the apex [12, 10], are solely due to the hydrodynamics of the tapered cochlea. Interestingly, at apical locations, the model also predicts the existence of "active gain" well above CF, a phenomenon recently observed in recordings from the apex of the guinea-pig cochlea [10].

References

[1] A. Altoè and C. A. Shera. Nonlinear cochlear mechanics without direct vibration-amplification feedback. *Phys. Rev. Res.*, 2:013218, 2020.

[2] L. H. Carney, M. J. McDuffy, and I. Shekhter. Frequency glides in the impulse responses of auditory-nerve fibers. *J. Acoust. Soc. Am*, 105:2384–2391, 1999.

[3] J. B. Dewey, B. E. Applegate, and J. S. Oghalai. Amplification and suppression of traveling waves along the mouse organ of Corti: Evidence for spatial variation in the longitudinal coupling of outer hair cell-generated forces. *J. Neurosci.*, 39:1805–1816, 2019.

[4] H. Duifhuis. Cochlear macromechanics. In G. M. Edelman, W. E. Gall, and W. M. Cowan, editors, *Auditory Function: Neurological Bases for Hearing*, pages 189–211. Wiley, New York, 1998.

[5] D. D. Greenwood. A cochlear frequency-position function for several species—29 years later. *J. Acoust. Soc. Am.*, 87:2592–2605, 1990.

[6] N. Y. S. Kiang, T. Watanabe, E. C. Thomas, and L. F. Clark. *Discharge Patterns of Single Fibers in the Cat's Auditory Nerve.* MIT, Cambridge, MA, 1965.

[7] H. Y. Lee, P. D. Raphael, A. Xia, J. Kim, N. Grillet, B. E. Applegate, A. K. E. Bowden, and J. S. Oghalai. Two-dimensional cochlear micromechanics measured in vivo demonstrate radial tuning within the mouse organ of Corti. *J. Neurosci.*, 36:8160–8173, 2016.

[8] M. C. Liberman. Auditory-nerve response from cats raised in a low-noise chamber. *J. Acoust. Soc. Am.*, 63:442–455, 1978.

[9] M. Müller. The cochlear place-frequency map of the adult and developing Mongolian gerbil. *Hear. Res.*, 94(1-2):148–156, 1996.

[10] A. Recio-Spinoso and J. S. Oghalai. Mechanical tuning and amplification within the apex of the guinea pig cochlea. *J. Physiol.*, 595:4549–4561, 2017.

[11] T. Ren, W. He, and D. T. Kemp. Reticular lamina and basilar membrane vibrations in living mouse cochleae. *Proc. Natl. Acad. Sci. USA*, 113:9910–9915, 2016.

[12] W. S. Rhode and N. P. Cooper. Nonlinear mechanics in the apical turn of the chinchilla cochlea in vivo. *Aud. Neurosci*, 3:101–121, 1996.

[13] L. Robles and M. A. Ruggero. Mechanics of the mammalian cochlea. *Physiol. Rev.*, 81:1305–1352, 2001.

[14] M. P. Scherer and A. W. Gummer. Impedance analysis of the organ of Corti with magnetically actuated probes. *Biophys. J.*, 87:1378–1391, 2004.

[15] R. A. Schmiedt. Spontaneous rates, thresholds and tuning of auditory-nerve fibers in the gerbil: Comparisons to cat data. *Hear. Res.*, 42(1):23–35, 1989.

[16] C. A. Shera. Laser amplification with a twist: Traveling-wave propagation and gain functions from throughout the cochlea. *J. Acoust. Soc. Am.*, 122: 2738–2758, 2007.

[17] C. A. Shera, J. J. Guinan, and A. J. Oxenham. Revised estimates of human cochlear tuning from otoacoustic and behavioral measurements. *Proc. Natl. Acad. Sci. USA*, 99:3318–3323, 2002.

[18] C. A. Shera, A. Tubis, and C. L. Talmadge. Do forward- and backward-traveling waves occur within the cochlea? Countering the critique of Nobili et al. *J. Assoc. Res. Otolaryngol.*, 5:349–359, 2004. A version correcting *JARO's* numerous typesetting errors is available at `https://apg.mechanicsofhearing.org`.

[19] C. A. Shera, A. Tubis, and C. L. Talmadge. Coherent reflection in a two-dimensional cochlea: Short-wave versus long-wave scattering in the generation of reflection-source otoacoustic emissions. *J. Acoust. Soc. Am.*, 118:287–313, 2005.

[20] C. A. Shera, J. J. Guinan, and A. J. Oxenham. Otoacoustic estimation of cochlear tuning: Validation in the chinchilla. *J. Assoc. Res. Otolaryngol.*, 11:343–365, 2010.

[21] M. van der Heijden and C. P. Versteegh. Energy flux in the cochlea: Evidence against power amplification of the traveling wave. *J. Assoc. Res. Otolaryngol.*, 16:581–597, 2015.

[22] L. Watts. *Cochlear mechanics: Analysis and analog VLSI*. PhD thesis, California Institute of Technology, 1993.

[23] L. Watts. The mode-coupling Liouville–Green approximation for a two-dimensional cochlear model. *J. Acoust. Soc. Am*, 108:2266–2271, 2000.

[24] G. Zweig. Linear cochlear mechanics. *J. Acoust. Soc. Am.*, 138:1102–1121, 2015.
